# Priapism during transurethral surgery under spinal anaesthesia: Implications and review of management options

**DOI:** 10.4103/0019-5049.72654

**Published:** 2010

**Authors:** Jyotirmoy Das, Achyut Deuri, Preety Mittal Roy, Vijaya Pant

**Affiliations:** Department of Anaesthesia, Pain Management and Perioperative Medicine, Fortis Hospital, Shalimar Bagh, New Delhi, India; 1 Department of Anaesthesiology, Pandit MM Malviya Hospital, New Delhi, India

Sir,

Priapism after neuraxial or general anaesthesia is rare and may delay or even cancel the planned urological procedure. Our patient was a 66-year-old gentleman with hypertension, diabetes, coronary artery disease (double vessel disease, anterolateral wall myocardial infarction 2 years back), and chronic obstructive pulmonary disease with benign prostatic hypertrophy (70 g prostate). He was posted for LASER prostatectomy under spinal anaesthesia in view of coexisting diseases. Level of block was T10 dermatome. Thirty minutes into the surgical procedure, the patient started having penile engorgement which became maximal over the next 10 min forcing us to stop the surgery. After achieving haemostasis and waiting for 15 min in hope of spontaneous detumescence, intravenous glycopyrrolate 0.2 mg followed by incremental doses of ketamine to a total of 50 mg was given. Throughout this period, patient was relaxed and pain free. Intracavernous injection of agonists was decided against in view of his cardiovascular status. After 1 hr of waiting and informing patient and attendants, further surgery was called off. Gradual spontaneous detumescence was observed in the third postoperative hour.

Intraoperative penile erection when observed is more common in patients younger than 50 years, with epidural anaesthesia or general anaesthesia with propofol.[1] It is difficult to perform transurethral procedure during penile erection because attempts to do so may lead to complications, such as excessive bleeding and urethral trauma.

The commonly quoted techniques for treatment of penile erection under anaesthesia are intravenous ketamine, glycopyrrolate and terbutaline;[2] increasing the depth of anaesthesia with inhalational anaesthetics; intracavernous injection of agonist (epinephrine,[3] Phenylephrine[1]) and dorsal nerve block. Intravenous glycopyrrolate was shown to be an effective drug especially because of its stable cardiovascular profile.[4]

Imbalance between sympathetic and parasympathetic nervous systems is considered as an underlying mechanism for intraoperative erection, although local stimulation before complete sensory blockade can contribute to the problem. Detumescence is mediated by adrenergic stimulation that causes a constriction of penile venous sinusoids and opening of emissary veins leading to increased blood drainage.[5] Psychogenic and reflex erections may occur during the early stages of spinal anaesthesia when the pathways involved are still incompletely blocked.[6]

Therapy must be quickly initiated to enhance venous drainage of the engorged corpora cavernosa before prolonged venous stasis leads to increased viscosity associated with slugging and impairment of the routes of venous egress.[7] It must be emphasized that for the successful detumescence of the penis, the relationship of treatment to the duration of erection is the critical factor and therapy should be tailored to the patient’s condition.

## References

[1] Staerman F, Nouri M, Coeurdacier P, Cipolla B, Guille F, Lobel B (1995). Treatment of the Intraoperative Penile Erection with Intracavernous Phenylephrine. J Urol.

[2] Shantha TR (1989). Intraoperative management of penile erection by using terbutaline. Anesthesiology.

[3] Mels F, van Driel JJ, Mensink KA (1991). Treatment of priapism by injection of adrenaline into the corpora cavernosa penis. Scand J Urol Nephrol.

[4] Valley, Marc A, Sang, Christine N (1994). Use of glycopyrrolate to treat intraoperative penile erection: Case report and review of the literature. Reg Anesth Pain Med.

[5] Bosch RJ, Benard F, Aboseif SR, Stief CG, Lue TF, Tanagho EA (1991). Penile detumescenece; characterization of three phases. J Urol.

[6] Bors I, Coman AI (1981). Neurological distribution of sexual dysfunction with special references in 529 patients with spinal cord injury. Urol Survey.

[7] Baltogiannis DM, Charalabopoulos AK, Giannakopoulos XK, Giannakis DJ, Sofikitis NV, Charalabopoulos KA (2006). Penile erection during transurethral surgery. J Androl.

